# Approach to Tuning the Dispersion Stability of TEMPO‐substituted Polymer Nanoparticles for Aqueous Organic Redox Flow Batteries

**DOI:** 10.1002/cssc.202500911

**Published:** 2025-08-10

**Authors:** Kohei Ishigami, Shinjiro Mori, Kenichi Oyaizu

**Affiliations:** ^1^ Department of Applied Chemistry Waseda University 3‐4‐1 Okubo, Shinjuku‐ku Tokyo 169‐8555 Japan; ^2^ Research Institute for Science and Engineering Waseda University 3‐4‐1 Okubo, Shinjuku‐ku Tokyo 169‐8555 Japan

**Keywords:** copolymer, organic redox flow batteries, redox‐active polymer nanoparticles, surface modification, zwitterions

## Abstract

Hydrophilic redox polymer nanoparticles with zwitterionic moieties are synthesized to improve material utilization for semisolid redox flow batteries (RFBs). TEMPO is chosen as the charge storage moiety, taking advantage of its high redox‐activity in pH‐neutral aqueous electrolytes. Redox‐active polymer nanoparticles copolymerized with the zwitterionic moiety show significant changes in surface properties, indicating promising dispersion stability and electrochemical performance even at more than 1 mol% zwitterionic moiety in the copolymer in prototype semisolid RFBs. Among the compositions studied, the introduction of 10 mol% zwitterionic moiety results in the best combination of material utilization and cycle stability. This approach is an effective molecular design strategy to achieve high performance and high volumetric density semisolid RFBs.

## Graphical abstract

This study presents zwitterion‐functionalized redox‐active polymer nanoparticles that enable stable dispersion and enhanced electrochemical performance in aqueous redox flow batteries (RFBs). 10 mol% zwitterionic content significantly improves material utilization and reversibility. The strategy maximizes redox efficiency in semisolid systems and highlights a promising route for high‐density, aqueous organic RFBs.

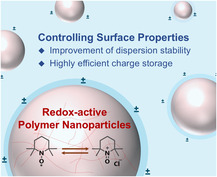

## 1 Introduction

**Figure 1:**
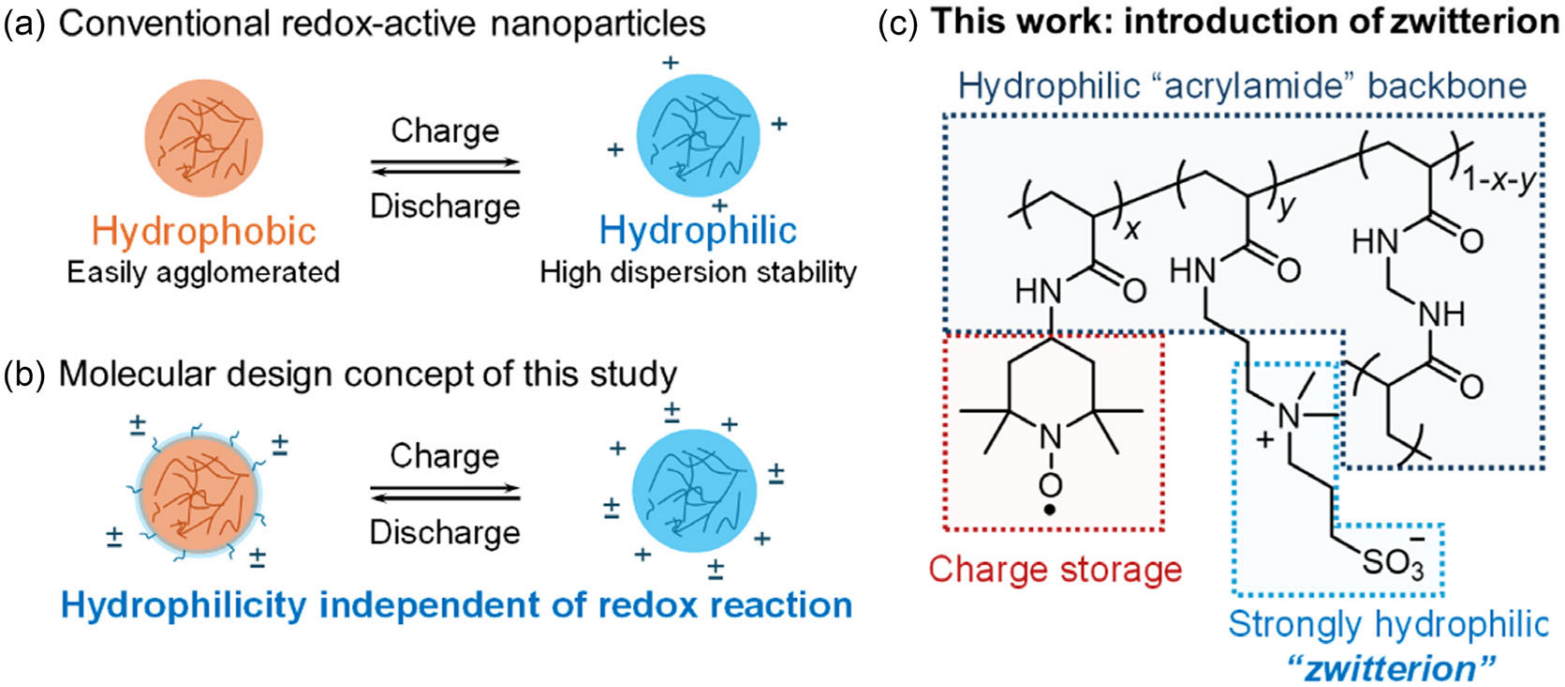
Molecular design concept of highly dispersible redox‐active polymer particles: a) Schematic representation of conventional redox‐active nanoparticles, which switch the hydrophilic/hydrophobic balance via a redox reaction. b) Molecular design strategy to achieve redox‐independent hydrophilicity. c) The structure of PTAm with zwitterionic moiety (This work).

Redox flow batteries (RFBs) with organic active materials have attracted attention as one of the promising candidates for grid‐scale energy storage in solutions.^[^ ^1^, ^2^, ^3^, ^4^, ^5^, ^6^ ^]^ Organic redox‐active molecules are recognized for their remarkable electrochemical properties based on flexible molecular tuning and fast electron transfer, in addition to their low cost and slight price fluctuations due to their use of rare metal‐free general chemicals as raw materials.^[^ ^1^, ^7^ ^]^ Certain organic‐based redox molecules, such as ferrocene, viologen, and TEMPO, exhibit excellent redox response in pH‐neutral aqueous electrolytes and may help reduce environmental impact when used as active materials in RFBs.^[^ ^3^, ^8^, ^9^ ^]^ Polymeric active materials with densely substituted redox moieties provide effective crossover inhibition while maintaining sufficient electrochemical properties for use in RFBs.^[^ ^10^, ^11^, ^12^ ^]^ In particular, polymers substituted with stable radicals such as TEMPO are expected as charge storage materials for RFBs due to their fast charge‐discharge properties based on self‐electron exchange reactions.^[^ ^6^, ^13^, ^14^ ^]^ However, the maximum volumetric energy density of the polymeric RFBs is determined by the upper limit of solubility. The development of polymeric active materials with high solubility is an important ongoing challenge.^[^ ^15^ ^]^ As an alternative approach, semisolid RFBs with insoluble but dispersible materials have been proposed to overcome the solubility limit.^[^ ^16^ ^]^ Examples include suspension and slurry types^[^ ^17^, ^18^, ^19^ ^]^ and redox targeting flow batteries that combine solid charge storage materials with dissolved charge transport materials.^[^ ^20^, ^21^, ^22^, ^23^ ^]^ Poly(TEMPO‐substituted acrylamide) (PTAm) is one of the hydrophilic radical polymers, which shows both moderate swelling and dispersion properties.^[^ ^24^, ^25^, ^26^ ^]^ PTAm is a rare example of a semisolid RFBs in pH‐neutral aqueous electrolytes operating at concentrations higher than 1.5 M (40 Ah L^−1^).^[^ ^19^ ^]^ Although acrylamide is used as the hydrophilic backbone, the hydrophobicity of TEMPO still dominates the overall property. Hydrophilic and hydrophobic switching in response to the redox reaction can also cause unwanted aggregation and sedimentation during charging and discharging (**Figure** **1**a). Improving the material utilization of semisolid RFBs is one of the most important issues.^[^ ^27^ ^]^ Material utilization is often evaluated in terms of the theoretical capacity ratio, which is typically less than 35% for polymer‐based semisolid RFBs.^[^ ^17^, ^18^, ^19^ ^]^ Conventional approaches have focused on improving dispersion stability by incorporating self‐assembly and/or adding surfactants.^[^ ^28^, ^29^, ^30^ ^]^ Surface modification with zwitterions is one of the useful techniques for maintaining the dispersion stability of inorganic particles in solutions with high ionic strength.^[^ ^31^, ^32^ ^]^ A robust hydration framework inhibits both particle contact and aggregation, even in highly concentrated salt solutions where electrostatic repulsion is less dominant. It is a useful strategy in RFB systems that require constant supporting electrolytes. In this study, PTAm nanoparticles copolymerized with a small amount of zwitterionic moieties were synthesized as a new approach to more effectively exploit the potential of active materials. It is designed to be highly and consistently hydrophilic, regardless of its redox state, due to the incorporated zwitterions (Figure 1b). Sulfobetaine was selected as the zwitterion (Figure 1c), which has been widely studied in the surface modification of biomaterials. The dispersion polymerization and oxidation reaction were carried out in the aqueous media with the intention that the highly hydrophilic sulfobetaine in the particles would face outward^[^ ^33^ ^]^ and the particle surface would be covered with a hydration layer when the redox‐active particles were stabilized. The surface state changed not only with the introduction of sulfobetaine, but also with charging and discharging. Both excellent cycle stability and material utilization were achieved with 10 mol% of sulfobetaine. This article demonstrates the high availability of polymeric semisolid RFBs, where low material utilization has always been an issue. The present approach is a universal strategy that can be applied to improve the stability and performance of batteries using redox‐active polymer nanoparticles.

## 2 Results and Discussion

### 2.1 Synthesis and Characterization of Redox‐active Nanoparticles

Crosslinked PTAm nanoparticles with different compositions of the zwitterionic moiety were designed to improve the dispersion stability by introducing the highly hydrophilic zwitterionic unit into the nanoparticles. The sulfobetaine‐substituted acrylamide comonomer with a feed ratio of 0≈10 mol% with respect to the piperidinyl acrylamide was used in copolymerization. The resulting copolymer, 2,2,6,6‐tetramethylpiperidyl‐4‐acrylamide, was obtained in a high yield (>90%) with high purity in a short time, simply by dropping acryloyl chloride in dichloromethane without the use of conventionally used bases or high‐boiling‐point organic solvents.^[^ ^25^, ^34^ ^]^ The capture of hydrogen chloride by the piperidine moiety eliminated the need for a base during the nucleophilic acyl substitution reaction. High scalability of more than 20 g was also achieved by appropriate management of the exothermic reaction. The monomer structure was identified by ^1^H‐NMR, ^13^C‐NMR, and atmospheric pressure chemical ionization mass spectrometry (APCI‐MS) (Figure S1 and S2, Supporting Information).

**Scheme 1:**
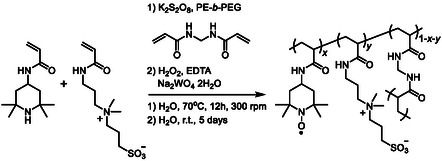
Synthesis of PTAm nanoparticles in this study.

TEMPO‐substituted polymer nanoparticles were synthesized via dispersion polymerization^[^ ^19^ ^]^ followed by the oxidation of the piperidine unit, similar to that for the conventional TEMPO‐substituted polymers (**Scheme** **1**). *N*,*N*‐methylenebisacrylamide was selected as the crosslinker. The feed ratio of the bifunctional monomer, i.e., the crosslinking density, was fixed at 6 mol%. It was difficult to estimate from IR the amount of zwitterion introduced in a small amount. Attempts to determine the zwitterion amount with ^1^H‐NMR for a model copolymer containing *N*‐isopropylacrylamide as a probe in place of the crosslinking agent was unsuccessful, due to the very low solubility of the model copolymer as a result of the small content of the zwitterion moiety. The dispersant was not removed in the oxidation reaction to maximize the effect of the dispersion stability. The structure of the dried polymer nanoparticles was determined by infrared spectroscopy (Figure S3–S7, Supporting Information). The absorption peak for the S=O stretching vibration (around 1038 cm^−1^) of the sulfonic acid appeared and increased with the feed ratio of the sulfobetaine comonomer with respect to the C=O stretching vibration derived from acrylamide. Nitroxide radical density was estimated from magnetic susceptibility measurements by VSM‐SQUID. The radical content was ≈80 mol%, calculated with respect to the total molar amount of piperidine units incorporated during polymerization (Figure S8, Supporting Information, **Table** **1**). Sulfobetaine moieties remained thermally stable at high temperatures without fragmentation. The 5% weight loss temperatures (*T*_d5%_) were higher than 255 °C, comparable to that of conventional PTAm (Figure S9, Supporting Information, Table 1).^[^ ^26^ ^]^

**Table 1:** Results of synthesis for PTAm nanoparticles.

Entry	Feed Ratio		Total yield [%]	Radical [%]a)	*T*_d5%_ [°C]b)
	*x* [mol%]	*y* [mol%]			
1	94	0.0	91	76	255
2	93	1.0	85	88	256
3	91	3.0	87	78	256
4	89	5.0	88	84	255
5	84	10	70	76	266

a) Determined by VSM‐SQUID.

b) Estimated by TGA.

### 2.2 Dispersion Stability in Aqueous Electrolytes

PTAm nanoparticles without zwitterionic moieties exhibit limited dispersion stability. Clustering of the nanoparticles with a small amount of the zwitterionic moiety was observed to stabilize the dispersed state. Above 5 mol% sulfobetaine, the dispersion maintained the initial particle size (**Table** **2**). PTAm nanoparticles exhibited a unimodal size distribution in both pure water and an electrolyte solution, and no apparent aggregation was observed.

**Table 2:** Particle size and diffusional properties of PTAm nanoparticles in aqueous solution with/without salts estimated by DLS.

Entry	Pure water		0.1 M NaCl (0 h)		0.1 M NaCl (24 h)	
	*d* (10^2^ nm)	log *D*_phys_ [–]	*d* (10^2^ nm)	log *D*_phys_ [–]	*d* (10^2^ nm)	log *D*_phys_ [–]
1	3.2	−7.8	3.3	−7.8	−a)	−a)
2	5.3	−7.9	3.8	−7.9	16	−8.5
3	5.7	−8.0	4.2	−7.9	12	−8.1
4	5.9	−8.0	3.5	−7.8	5.6	−7.9
5	6.2	−8.0	6.0	−8.0	6.3	−7.9

a) Significant sedimentation.

Particle size in aqueous solution was estimated by dynamic light scattering (DLS), and physical diffusion coefficients (*D*_phys_) were calculated using the Stokes‐Einstein equation, assuming a spherical shape. In pure water, the particle size seemed to increase stepwise with the introduction of the sulfobetaine unit (Figure S10, Supporting Information), which should be attributed to the formation of a hydration layer on the particle surface and swelling of the polymer chains. To clarify the extent of this size increase, the original particle size was also evaluated by transmission electron microscopy (TEM) imaging (Figure S11, Supporting Information), which revealed diameters of ≈300 nm for all compositions. Upon hydration, the IR spectra exhibited characteristic shifts in the amide regions: the C=O stretching band redshifted, while the N—H stretching band blueshifted (Figure S12, Supporting Information). These spectral changes suggest that intermolecular hydrogen bonding among amide groups in the dry state is replaced with hydrogen bonding between water and the carbonyl groups, accompanied by the emergence of less‐restricted N—H groups.^[^ ^35^ ^]^ A slight blue shift in the S=O stretching band was observed only at higher zwitterionic content (e.g., 10 mol%), suggesting that intramolecular electrostatic interactions between the sulfonate and ammonium groups are weakened and replaced by watermediated hydration, altering the local bonding environment.^[^ ^36^ ^]^ These observations indicate that, in aqueous environments, polymer nanoparticles undergo structural reorganization driven by hydrogen bonding and electrostatic shielding. However, the hydration‐induced spectral changes associated with the zwitterionic groups become discernible only when a sufficient amount of sulfobetaine is incorporated. These molecular‐level observations support the interpretation that the increase in DLS‐measured size arises not from aggregation, but from hydration‐layer formation and polymer swelling. This behavior highlights the critical role of zwitterionic moieties in structuring the nanoparticle surface in water conditions.

**Figure 2:**
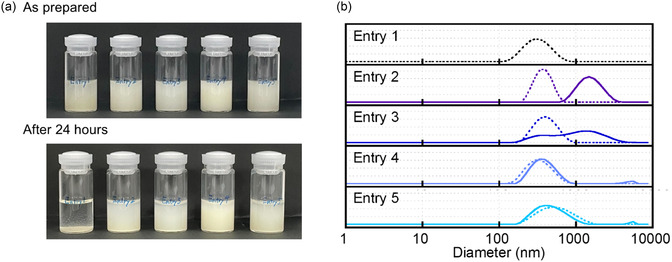
Evaluation of dispersion stability of the PTAm nanoparticles in 0.1 M NaCl aqueous solution: a) Photograph of each dispersion, arranged from left to right by Entries 1–5. b) Their DLS distribution. The dashed curve is obtained as prepared, and the solid line is after 24 h. Entry 1 was sedimented and no data was available after 24 h.

0.1 M NaCl aqueous solution was selected as the electrolyte solution. In high‐concentration electrolytes, polymer nanoparticles are known to dehydrate and shrink, causing aggregation, sedimentation, and reduced redox accessibility due to saltation.^[^ ^27^ ^]^ In the electrolyte solution, the zwitterionic charge on the surface of the nanoparticle was shielded by the external sodium and chloride ions, causing the polymer chains to shrink. Incorporation of 10 mol% sulfobetaine led to a reduction in the shrinkage, due to the stable surface structure already formed by the hydration layer. Dispersion stability was assessed by standing the suspension for 24 h (**Figure** **2**a). Significant sedimentation was observed for the nanoparticle obtained with entry 1 without sulfobetaine. As a result, no data was available after 24 h. Interestingly, the nanoparticles for entries 2 and 3 containing low amounts of sulfobetaine formed stable clusters through partial aggregation. In particular, the nanoparticles in entry 2 (1 mol%) appeared to achieve metastable dispersion stability via aggregation‐based clustering, rather than hydration‐layer stabilization. Entry 3 (3 mol%) represented a transitional state, in which clustering and hydration‐layer formation competed, resulting in insufficient stabilization and relatively unstable dispersion. These clusters were likely caused by insufficient zwitterionic density to fully stabilize the individual nanoparticles. In contrast, nanoparticles containing 5 mol% or more sulfobetaine maintained their initial particle size distribution over 24 h. While the overall particle size appeared larger due to minor peaks corresponding to aggregates, the main peak remained consistent with the initial distribution. The mechanism of stabilization of the dispersed state depends on the amount of zwitterionic moiety introduced into the nanoparticle. The effect of the zwitterionic moiety on stabilization was evident even at low loadings, with only 5 mol% sulfobetaine fully maintaining the original particle size over 24 h. This approach offers a broadly applicable strategy for stabilizing redox‐active nanoparticles, where colloidal stability must be achieved without compromising functional performance.

### 2.3 Electrochemical Behavior of Redox‐active Polymer Nanoparticles

**Figure 3:**
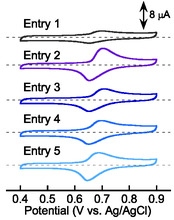
Cyclic voltammogram of 5 mM particle dispersions in 0.1 M NaCl aqueous solution, scanned at 50 mV s^−1^.

Cyclic voltammetry obtained for the nanoparticles dispersed in 0.1 M NaCl aqueous solution showed a redox response at *E*_1/2_ = 0.67≈0.68 V versus. Ag/AgCl, independent of the copolymer composition (**Figure** **3**, Figure S13, Supporting Information). This was comparable to the electrochemical response obtained for the conventional TEMPO‐substituted polymers.^[^ ^6^, ^8^, ^24^, ^25^, ^34^ ^]^ The dispersions for entries 2≈5 demonstrated higher peak current values and thus higher concentrations of the TEMPO groups involved in the electrode reaction compared to the dispersion for entry 1, as a result of the high dispersion stability (Figure 3). A small amount of the nanoparticles for entry 1 involved in the reaction were observed to show adsorption behavior on the glassy carbon electrode. The apparent diffusion coefficients for charge transport (*D*_app_) estimated by chronoamperometry and the standard electrochemical reaction constant (*k*_0_) determined by Nicholson's method were generally comparable to those for the previously reported active materials for colloidal and organic semisolid RFBs (Figure S14, Table S1, Supporting Information).^[^ ^18^, ^19^, ^37^, ^38^ ^]^ The ratio of *D*_app_/*D*_phys_ was less than 10^3^, which was within a reasonable range for polymer nanoparticles.^[^ ^19^ ^]^

### 2.4 Battery Test and Surface Properties During Charge‐discharge

**Figure 4:**
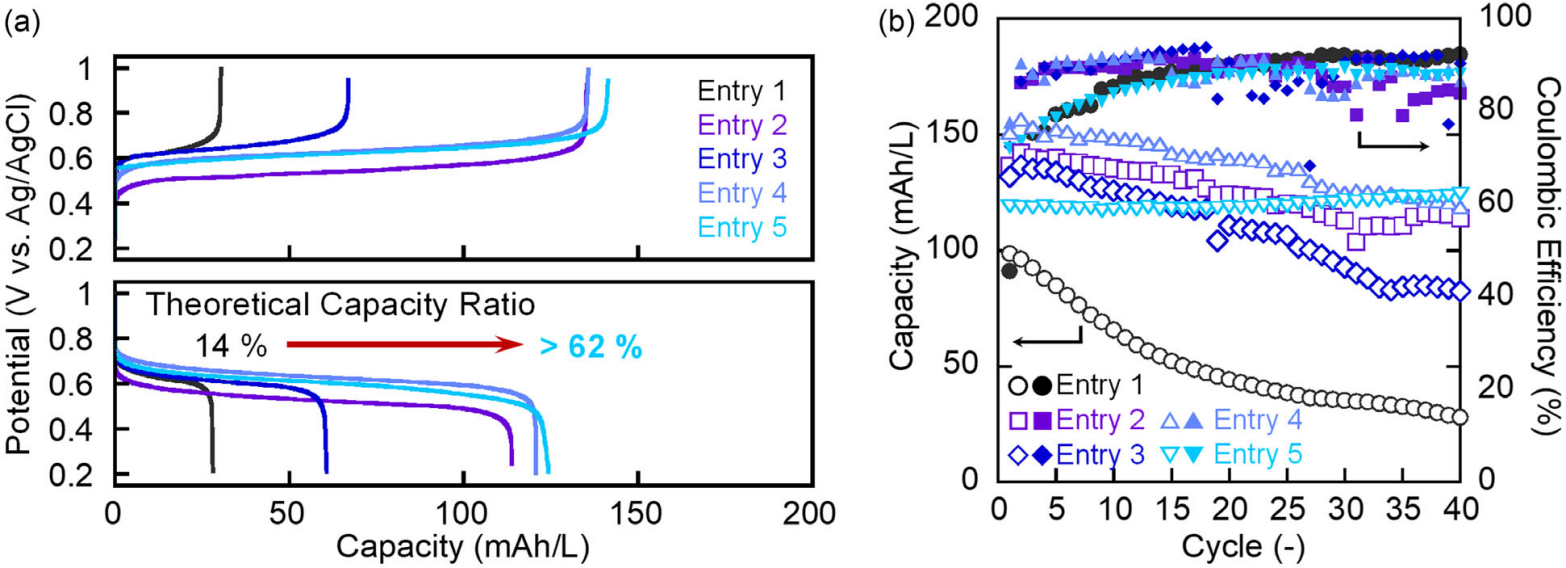
Charge‐discharge properties of each particle (0.1 C, theoretical capacity: 200 mAh L^−1^): a) Charge‐discharge curve at 40th cycle. b) Cycle performance.

Charge and discharge characteristics of the polymer nanoparticles were evaluated using an Htype cell (**Figure** **4**). To focus on the effects of the zwitterionic moieties in the polymer nanoparticles, the counter side of the cell was charged with a solution of ethyl viologen dichloride, and an anion‐exchange membrane was used as the separator. All zwitterion‐containing samples (entries 2≈5) displayed markedly improved cycling stability compared to entry 1. Cycling stability was higher for the nanoparticles in entries 2≈5 with a small amount of sulfobetaine than that in entry 1. Among them, entry 5 (10 mol %) exhibited the best long‐term performance, showing no detectable capacity fade over 40 cycles and retaining 64% of its theoretical capacity. Entry 4 (5 mol%) delivered the highest initial capacity but began to decline after 20 cycles and was eventually surpassed by entry 5. Entry 2 (1 mol%) attained moderate stability through aggregation‐based clustering, whereas entry 3 (3 mol%) represented a transitional regime in which clustering and hydration‐layer formation competed, leading to insufficient stabilization and inferior retention. These trends confirm that the zwitterionic moiety stabilizes the dispersion state, and that a hydration‐layer‐dominated mechanism becomes fully effective at more than 5 mol%, culminating in the exceptional durability of the 10 mol% formulation.

**Figure 5:**
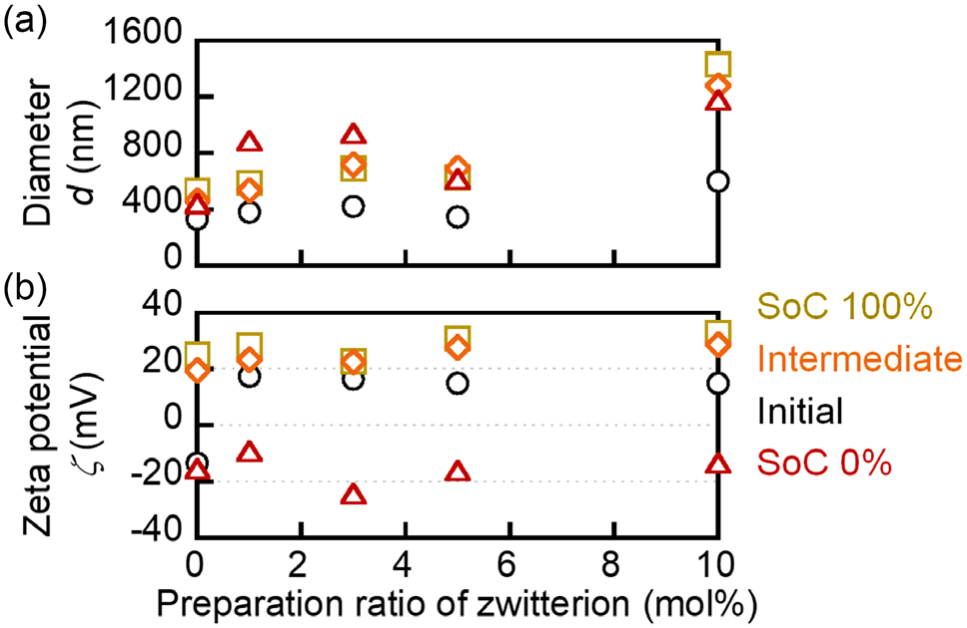
Surface properties of polymer nanoparticles in response to charge/discharge. The intermediate state corresponds to SoC 60% for Entries 1–3, SoC 40% for Entry 4, and SoC 43% for Entry 5, reflecting variations in theoretical capacity: a) Diameter of nanoparticles, estimated by DLS. b) Zeta potential diluted to 10 mM NaCl aqueous solution (pH 7), estimated by ELS.

The particle size and the zeta potential were evaluated during the 3rd cycle of discharge to analyze the surface properties of the PTAm particles (**Figure** **5** and Figure S15–S19, Table S2, Supporting Information). The Initial state was defined as immediately after the preparation of the dispersion, while 100% state of charge (SoC) corresponded to the point at which the upper cutoff potential (1 V vs. Ag/AgCl) was reached. An increasing trend in the zeta potential upon charging was consistently observed for all samples.

Entry 1, without sulfobetaine, exhibited a negative initial zeta potential due to sulfate groups introduced from the potassium persulfate initiator.^[^ ^39^ ^]^ The hydrated chloride anions incorporated during charging were not strongly retained, allowing reversible changes in both particle size and zeta potential upon discharge. In contrast, entries 2 ≈ 5 containing sulfobetaine exhibited a reversal to positive zeta potential at the Initial state, indicating that zwitterionic moieties significantly alter surface properties and ionic interactions beyond their nominal incorporation ratio. Notably, the zeta potental of zwitterioniccontaining nanoparticles (entry 2≈5) showed a pronounced difference between the initial and SoC 0% states. This suggests that chloride anions taken up during charging are retained at the particle's surface even after discharge, which likely stabilized the hydration layer. Such surface‐level ionic trapping and hydration may alter the electrostatic environment, leading to distinct clustering behavior during redox cycling. The degree of the zeta potential shift based on the redox reaction was almost the same or even higher than those for previously reported redox‐active polymer micelles.^[^ ^40^ ^]^ Among zwitterion‐containing nanoparticles, a tendency for irreversible clustering was observed during the redox process, suggesting that redox reactions led to surface reorganization to induce cluster formation. In samples with the low zwitterion content (entries 2 and 3), it is likely that the clustering was triggered during discharge as the zeta potential approached 0 mV, leading to the loss of electrostatic repulsion and the emergence of hydrophobic interactions between the nanoparticles. In fact, no significant change in the particle size was observed at SoC of ≈ 60%, whereas a clear increase in the particle size was detected after full discharge, supporting the progress of the redox‐induced clustering. This interpretation was further evidenced by the similar clustering observed upon simply standing the particles under the fully discharged conditions (Figure 2b), suggesting that the aggregation was primarily driven by electrostatic instability caused by the small zeta potential near *ζ* ≈ 0 mV, rather than the redox cycling itself.

At 5 mol% of the zwitterion composition, the particle size remained stable throughout the charge and discharge cycles, likely due to sufficient surface hydration to prevent the nanoparticles from close approach even near *ζ* = 0 mV. At 10 mol%, this effect was more pronounced. Swollen clusters formed in advance exhibited slight expansion and contraction in response to redox changes, but no significant changes in size were observed. The nanoparticles with the sulfobetaine composition of 10 mol% improved in both redox reactivity and dispersion stability, yielding high‐performance polymer nanoparticles for semisolid RFBs. This strategy should offer a basis for future high‐concentration systems toward high‐energy‐density organic RFBs.

## 3 Conclusion

Redox‐active polymer nanoparticles incorporating zwitterionic comonomers were synthesized as active materials for aqueous organic RFBs operating under pH‐neutral conditions. The nanoparticles were designed to retain hydrophilicity independent of the redox state. The zwitterion composition was tuned between 1 and 10 mol%. Even small amounts of zwitterions significantly improved both high dispersion stability to show favorable electrochemical performance. Notably, 10 mol%, highly reversible and efficient charge‐discharge cycles were achieved without significant capacity decay due to enhanced dispersion stabilization by a strong hydration layer. This approach to polymer nanoparticles effectively maximizes the electrochemical potential of active materials and provides a robust design strategy for future high‐concentration semisolid RFBs. Eliminating the effect of a small amount of contaminated oxygen that is likely to have lowered the coulombic efficiency (< 90% along the cycling) should lead to better cycling performance, which is the topic of our continuous research.

## 4 Experimental Section

4.1

4.1.1

##### Materials

4 ‐Amino‐2,2,6,6‐tetramethylpiperidine, acryloyl chloride, *N*,*N*′‐methylenebisacrylamide, 3‐[(3‐acrylamidopropyl)dimethylammonio]propane‐1‐sulfonate, ethylenediaminetetraacetic acid were purchased from Tokyo Chemical Industry Co. Potassium peroxodisulfate, sodium tungstate dihydrate, sodium chloride, and the dehydrated organic solvents were purchased from Kanto Chemical Co. 30% Hydrogen peroxide aqueous solution and ultrapure water were purchased from FUJIFILM Wako Pure Chemical Corp. Ethylviologen dibromide and poly(ethylene‐*b*‐ethylene glycol) were purchased from Sigma‐Aldrich. Cellulose dialysis membrane with molecular weight cutoffs of 3500 and 14 000 was purchased from Viskase Co.

##### Electrochemical Measurement

A conventional potentiostat (ALS660D, BAS) was employed for electrochemical measurements. All electrodes were purchased from BAS. A conventional three‐electrode system was employed using a platinum coil with 0.5 mm in diameter as a counter electrode and an Ag/AgCl wire as a reference electrode. Ag/AgCl electrodes were calibrated to 0.196 V versus. SHE with a 3 M NaCl aqueous solution as the internal solution. A glassy carbon disk electrode with a diameter of 1.6 mm was used as a working electrode for cyclic voltammetry and chronoamperometry. The electrolyte for the voltammetry was a 0.1 M NaCl solution. Before measurement, all particles were dispersed in solutions for 15 min using ultrasonic homogenizer by Emerson Electric Co. (BRANSON 250‐Advanced). All measurements were performed at room temperature, near 25 °C.

##### Battery Test

A prototype cell was fabricated using the conventional H‐type cell (EC Frontier Co.). A carbon felt (0.6 × 3.3 cm with a thickness of 3 mm, EC Frontier Co.) was used as a current collector. Before use, they were hydrophilized by soaking them in a poly(ethylene‐*b*‐ethylene glycol) aqueous solution for one day. As a separator, an anionic exchange membrane (SELEMION, AGC Co.) was used. On the counter side, an electrolyte solution of ethylviologen dichloride was employed as the active material, which was purified by ion exchange resin treatment (Amberlite, IRA410J Cl, purchased from Organo Co.). All nanoparticles were converted to specific capacity per mass (mAh g^−1^) considering radical density. The volumetric theoretical capacity of nanoparticles for the prototype cell was unified at 200 mAh L^−1^. Ethyl viologen dichloride was prepared at 600 mAh L^−1^ as the counter electrolyte. The electrolyte for the battery tests was a 0.1 M NaCl solution. During charging and discharging, nitrogen was flowed through the counter electrode tank for up to 7 days, after which it was sealed with Teflon sealing tape and parafilm. During the measurement, the sample was constantly agitated to facilitate mass transfer.

##### Synthesis: Synthesis of 2,2,6,6‐tetramethylpiperidyl acrylamide

4‐Amino‐2,2,6,6‐tetramethylpiperidine (20 mL, 115 mmol) and dichloromethane (300 mL) were added to a round bottom flask. The mixture was cooled to 0 °C. Acryloyl chloride (9.4 mL, 115 mmol) was slowly added dropwise to the mixture. The nucleophilic acyl substitution reaction was allowed to proceed by stirring at room temperature for 1 h. The product was extracted with saturated sodium carbonate solution. The organic layer was dehydrated with sodium sulfate, filtered, and evaporated. Recrystallization with hexane and ethanol produced white crystals (yield: 91%, Figure S1 and S2, Supporting Information). ^1^H‐NMR (500 MHz, CDCl_3_) *δ*: 6.28 (dd, *J* = 16.7, 1.4 Hz, 1H, *α*‐vinyl), 6.06 (dd, *J* = 17.0, 10.2 Hz, 1H, *β*‐vinyl on the C=O side), 5.64 (dd, *J* = 10.2, 1.1 Hz, 1H, *β*‐vinyl on the terminal side), 5.36 (s, 1H, amide‐NH), 4.35 (s, 1H, CH of piperidine ring), 1.94 (dd, *J* = 12.8, 3.7 Hz, 2H, CH_2_ of piperidine ring), 1.27 (s, 6H, CH_3_), 1.13 (s, 6H, CH_3_), 0.96 (dd, *J* = 25.2, 13.3 Hz, 2H, CH_2_ of piperidine ring). ^13^C‐NMR (125 MHz, CDCl_3_) *δ*: 164.4, 131.2, 126.4, 51.4, 45.4, 42.8, 35.1, 28.6. APCI‐MS (*m*/*z*): [M + H]^+^ = 211.18 (found), 211.33 (calc.). m.p.: 118 °C.

##### General Procedure for Synthesis of Poly(TEMPO‐substituted acrylamide) Nanoparticles

2,2,6,6‐tetramethylpiperidyl acrylamide, 3‐[(3‐acrylamidopropyl)dimethylammonio]propane‐1‐sulfonate (total of both monomers: 15.6 mmol), *N*,*N*’‐methylenebisacrylamide (0.15 g, 1 mmol), poly(ethylene‐*b*‐ethylene glycol) ([dispersant]/[monomer] = 0.30 (w/w)) and water (120 mL, total: 0.13 M) were added to a round bottom flask. Each condition of the monomer details is shown in Table 1. The mixture was heated to 70 °C for the dissolution of monomers and forcefully stirred at 300 rpm with a nitrogen atmosphere. After dissolution, potassium peroxodisulfate (32 mg, 0.12 mmol) was added to the mixture. The mixture was stirred at 300 rpm for 12 h at 70 °C. After dispersion polymerization, the mixture was cooled to room temperature. Ethylenediaminetetraacetic acid (40 mg, 0.14 mmol) and sodium tungstate dihydrate (192 mg, 0.58 mmol) were added to the flask. Hydrogen peroxide (15 mL, 146 mmol) was added in three equivalents every 24 h for a total of 5 days. Inorganic salts were removed by careful dialysis with distilled water for one week. After the removal of the solvent and vacuum drying, pale red to orange polymer nanoparticles were obtained. The PTAm nanoparticle without the zwitterionic comonomer (entry 1) was synthesized as previously reported.^[^ ^19^ ^]^

## Conflict of Interest

The authors declare no conflict of interest.

## Supporting information

Supplementary Material

## Data Availability

The data that support the findings of this study are available in the supplementary material of this article.
