# Supplementary material for: Approach to Tuning the Dispersion Stability of TEMPO‐substituted Polymer Nanoparticles for Aqueous Organic Redox Flow Batteries
Source: ChemSusChem. 2025 Aug 10;18(18):e202500911. doi: 10.1002/cssc.202500911 (PMC12456373; doi:10.1002/cssc.202500911)
Supplement: Supplementary file 1 — Supplementary Material [file CSSC-18-e202500911-s001.pdf]

Supplementary Information

Approach to Tuning the Dispersion Stability of TEMPO-substituted Polymer Nanoparticles for Aqueous Organic Redox Flow Batteries

Kohei Ishigami, [a] Shinjiro Mori, [a] and Kenichi Oyaizu *[a], [b]

[a] K. Ishigami, S. Mori, Prof. Dr. K. Oyaizu
Department of Applied Chemistry, Waseda University
3-4-1 Okubo, Shinjuku-ku, Tokyo 169-8555, Japan.
E-mail: oyaizu@waseda.jp
[b] Prof. Dr. K. Oyaizu
Research Institute for Science and Engineering, Waseda University
3-4-1 Okubo, Shinjuku-ku, Tokyo 169-8555, Japan.

Measurements

NMR spectra were recorded using JEOL-ECX500. Mass spectra were obtained by atmospheric-pressure chemical ionization (APCI) methods recorded with Bruker Compact. A Quantum Design MPMS SQUID-VSM magnetometer was used for magnetization measurements. Nanoparticle morphology was observed by transmission electron microscopy (TEM) using a Hitachi Hightech Corp. H-7650 operated at an accelerating voltage of 100 kV. Aqueous dispersions of polymer nanoparticles (1 mM) were drop-cast onto 150-mesh carbon-coated copper grids and vacuum-dried. Prior to observation, the dried grids were exposed to ruthenium (VIII) tetroxide vapor to enhance contrast. Infrared spectra were recorded using a JASCO FT/IR-4X spectrometer by both the KBr pellet method and ATR. The KBr pellet method was employed to confirm vibrational features in the dry state, while ATR-FTIR spectra were collected for both dry and hydrated samples to investigate hydration-induced shifts. Thermogravimetric analyses (TGA) were performed using Rigaku TG8120 under a nitrogen atmosphere. To eliminate the effect of moisture absorption, all samples were heated to 90°C at 5 °C min^{-1} and held for 1 hour. The temperature was then increased at 10 °C min^{-1}. Differential scanning calorimetry (DSC) were performed using TA Instruments Q200 at a scan rate of 10 °C min^{-1} under a nitrogen atmosphere for estimation of melting point of the piperidine-substituted monomer. Dynamic light scattering (DLS) and electrophoretic light scattering (ELS) were performed at near room temperature on a Malvern Panalytical Ltd. ZEN3600. ELS was conducted in 10 mM NaCl aqueous solution (pH = 7).

Estimation of electrochemical parameters

Determination of the apparent diffusion coefficient

The apparent diffusion coefficient D_{app} was determined by chronoamperometry using the Cottrell equation:

$$i\,(t) = \frac{nFAC\sqrt{D_{app}}}{\sqrt{\pi t}} \tag{1}$$

where: i (t) is the current (A) at time t (s), n is the number of electrons transferred, F is Faraday's constant (96485 C mol^{-1}), A is the electrode area (cm^2), C is the bulk concentration of the redox species (mol cm^{-1}), D_{app} is the apparent diffusion coefficient ($cm^2\ s^{-1}$). Chronoamperometric measurements were carried out using a three-electrode configuration with a glassy carbon disk electrode (φ = 1.6 mm, A = 0.0201 cm^2) as the working electrode, an Ag/AgCl reference electrode, and a platinum wire as the counter electrode. All experiments were conducted at room temperature near 25 °C. The potential was stepped from the open circuit potential to +1.0 V vs. Ag/AgCl, where the oxidation of the redox-active

nanoparticles was confirmed to proceed under diffusion-limited conditions. The electrolyte solution contained 5 mM of redox-active nanoparticles and 0.1 M NaCl as the supporting electrolyte.

Determination of the standard electrochemical reaction constant

The standard electrochemical reaction constant was estimated from cyclic voltammetry measurements using the Nicholson method for quasi-reversible systems.

$$\Psi = \frac{k_0}{\sqrt{\pi D f v}} \tag{2}$$

$$\Psi = \frac{-0.6288 + 0.0021\, n\Delta E_p}{1 - 0.017 n\Delta E_p} \tag{3}$$

where: ψ is the dimensionless Nicholson parameter, D is the diffusion coefficient ($cm^2\ s^{-1}$), $f = nF/RT$ constant combining the number of electrons n, Faraday's constant F, the gas constant R, and the temperature T. The peak separation ΔE_p was determined at each scan rate. The dimensionless Nicholson parameter ψ was evaluated using the widely used empirical approximation.

Calculation of Theoretical Capacity

The theoretical capacity, C_{theo} of the polymer was calculated using:

$$C_{theo} = \frac{nF}{M} \cdot \frac{1000}{3600} \tag{4}$$

where n is the number of electrons per redox unit (1 for TEMPO), F is Faraday's constant, and M is the molecular weight of the redox-active repeating unit (TEMPO-based). The effective specific capacity was adjusted by the radical content, z, estimated by magnetic susceptibility measurements (VSM-SQUID).

$$C_{eff} = C_{theo}\, z \tag{5}$$

Based on these values, sample concentrations were adjusted to achieve a consistent theoretical capacity of 200 mAh L^{-1} across all samples, irrespective of comonomer composition or zwitterion loading. The electrolyte volume and polymer mass were tailored accordingly for each formulation.

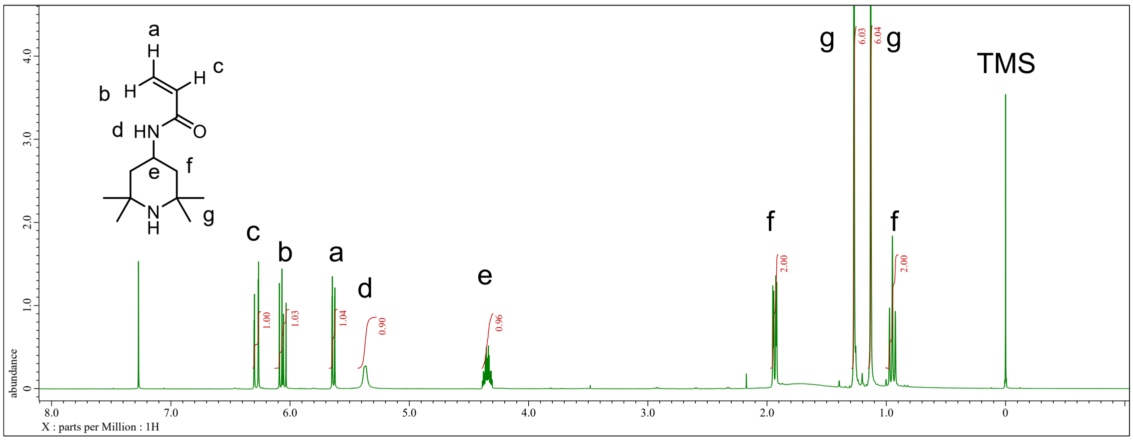

Figure S1. ^{1}H-NMR spectrum of piperidine-substituted acrylamide in $CDCl_3$.

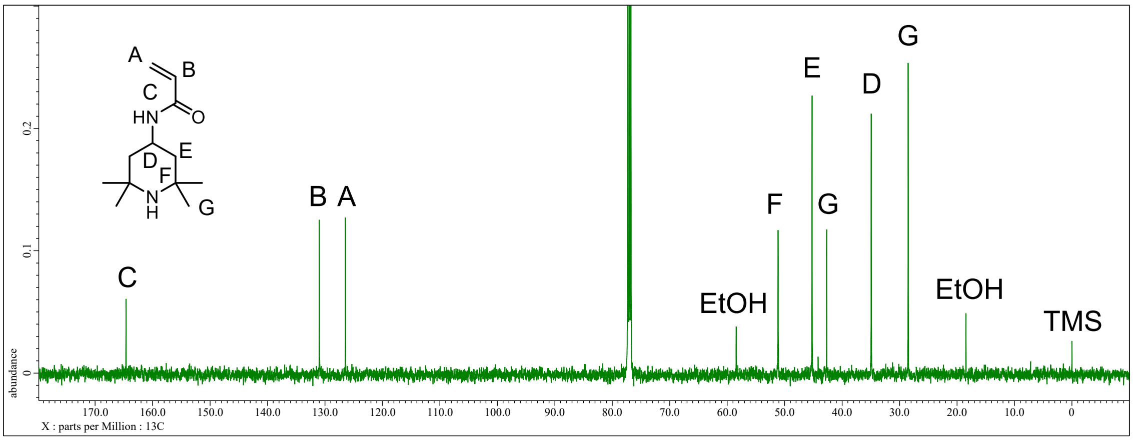

Figure S2. ^{13}C-NMR spectrum of piperidine-substituted acrylamide in $CDCl_3$.

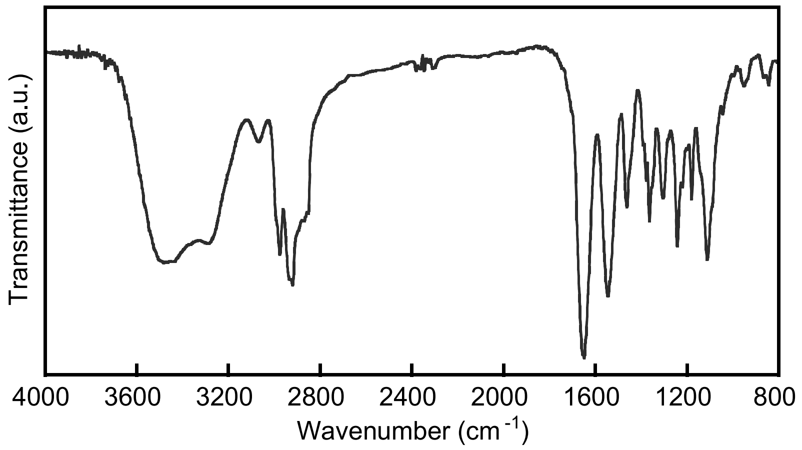

Figure S3. IR spectrum of poly(TEMPO-substituted acrylamide) nanoparticle (Entry 1).

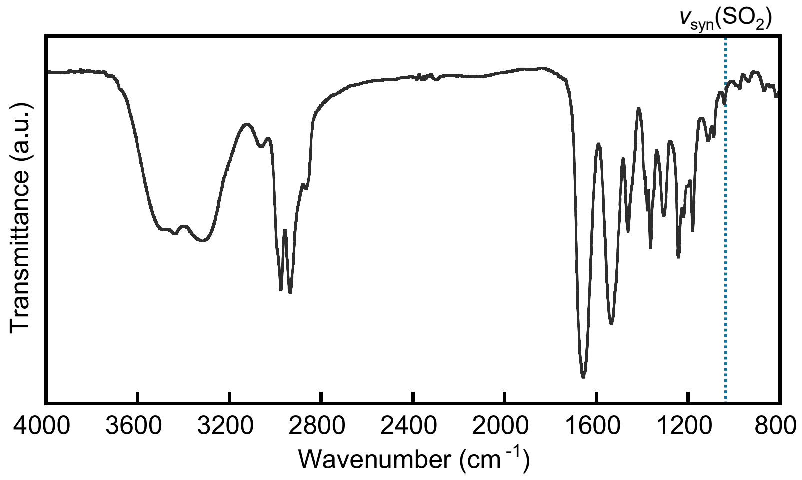

Figure S4. IR spectrum of poly(TEMPO-substituted acrylamide) nanoparticle (Entry 2).

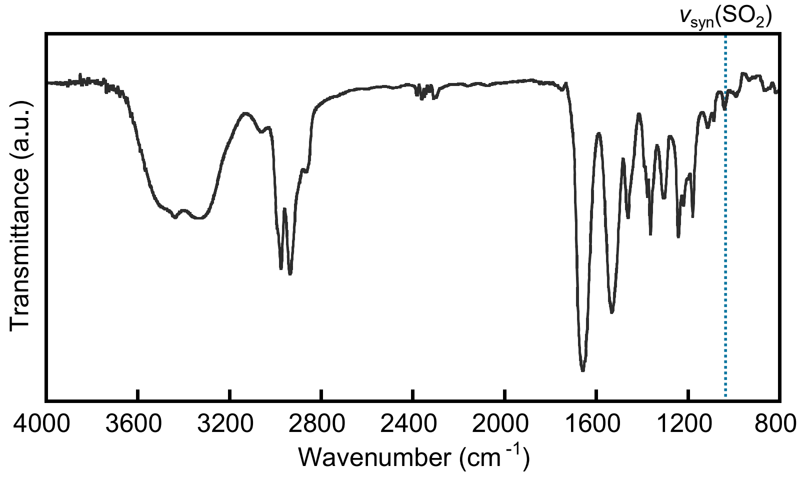

Figure S5. IR spectrum of poly(TEMPO-substituted acrylamide) nanoparticle (Entry 3).

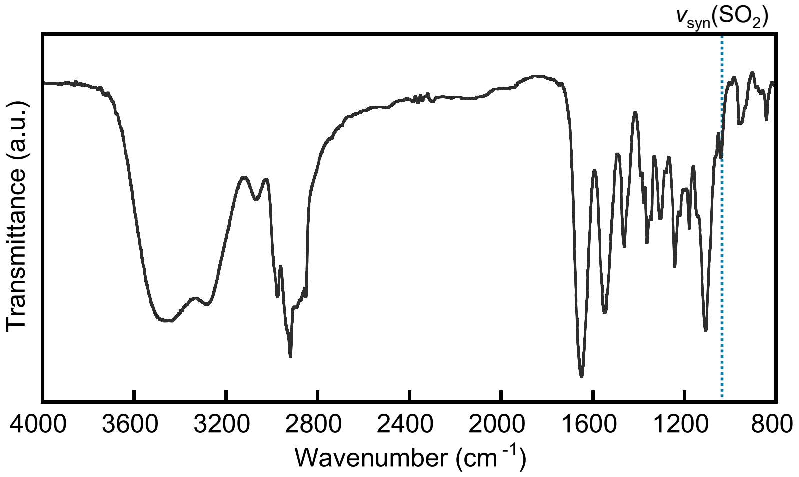

Figure S6. IR spectrum of poly(TEMPO-substituted acrylamide) nanoparticle (Entry 4).

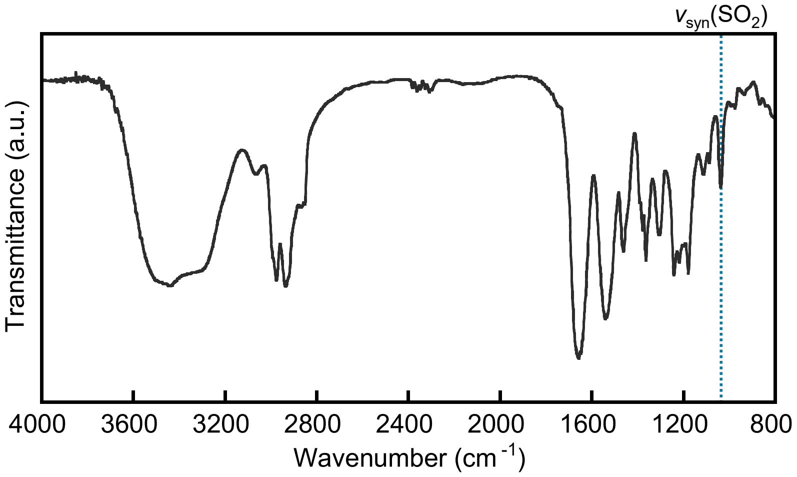

Figure S7. IR spectrum of poly(TEMPO-substituted acrylamide) nanoparticle (Entry 5).

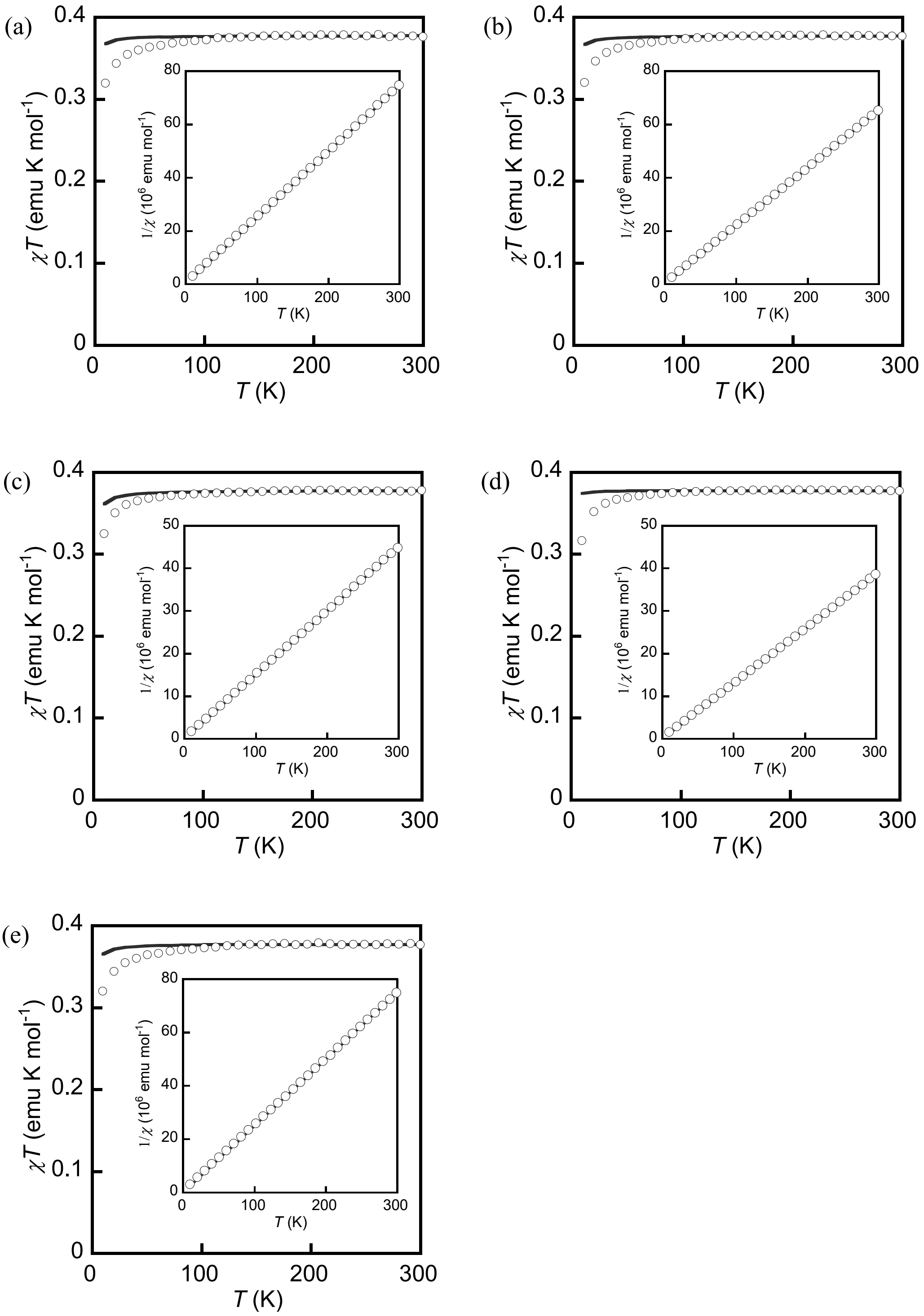

Figure S8 χT vs. T plots, inset: $1/\chi$ vs. T for poly(TEMPO-substituted acrylamide) nanoparticles obtained by SQUID measurements. (a) Entry 1, $y = 0.00$ (b) Entry 2, $y = 0.01$ (c) Entry 3, $y = 0.03$ (d) Entry 4, $y = 0.05$ and (e) Entry 5, $y = 0.10$.

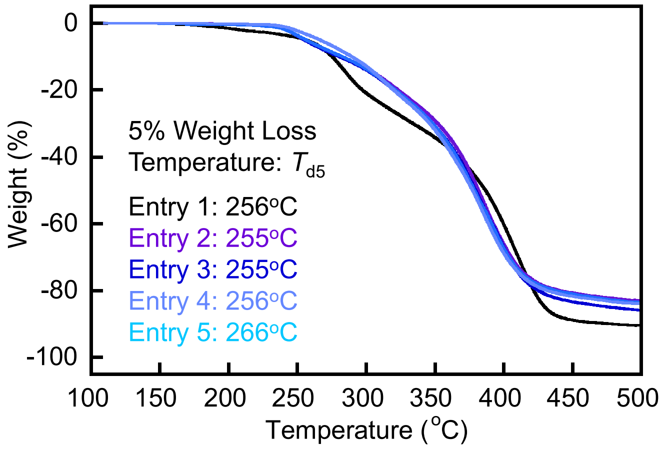

Figure S9 TGA traces, scanned at 10°C/min.

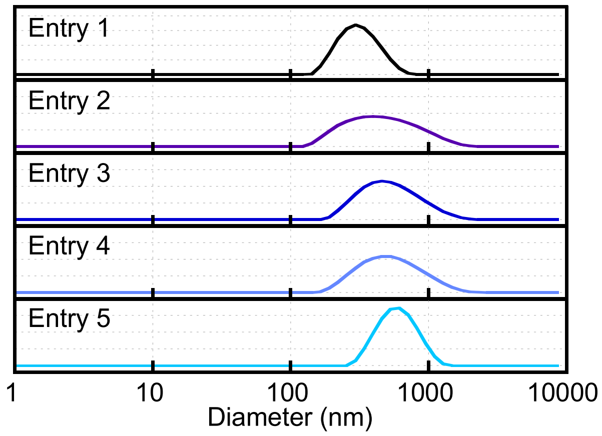

Figure S10 DLS distribution of each particle in pure water at 25 °C

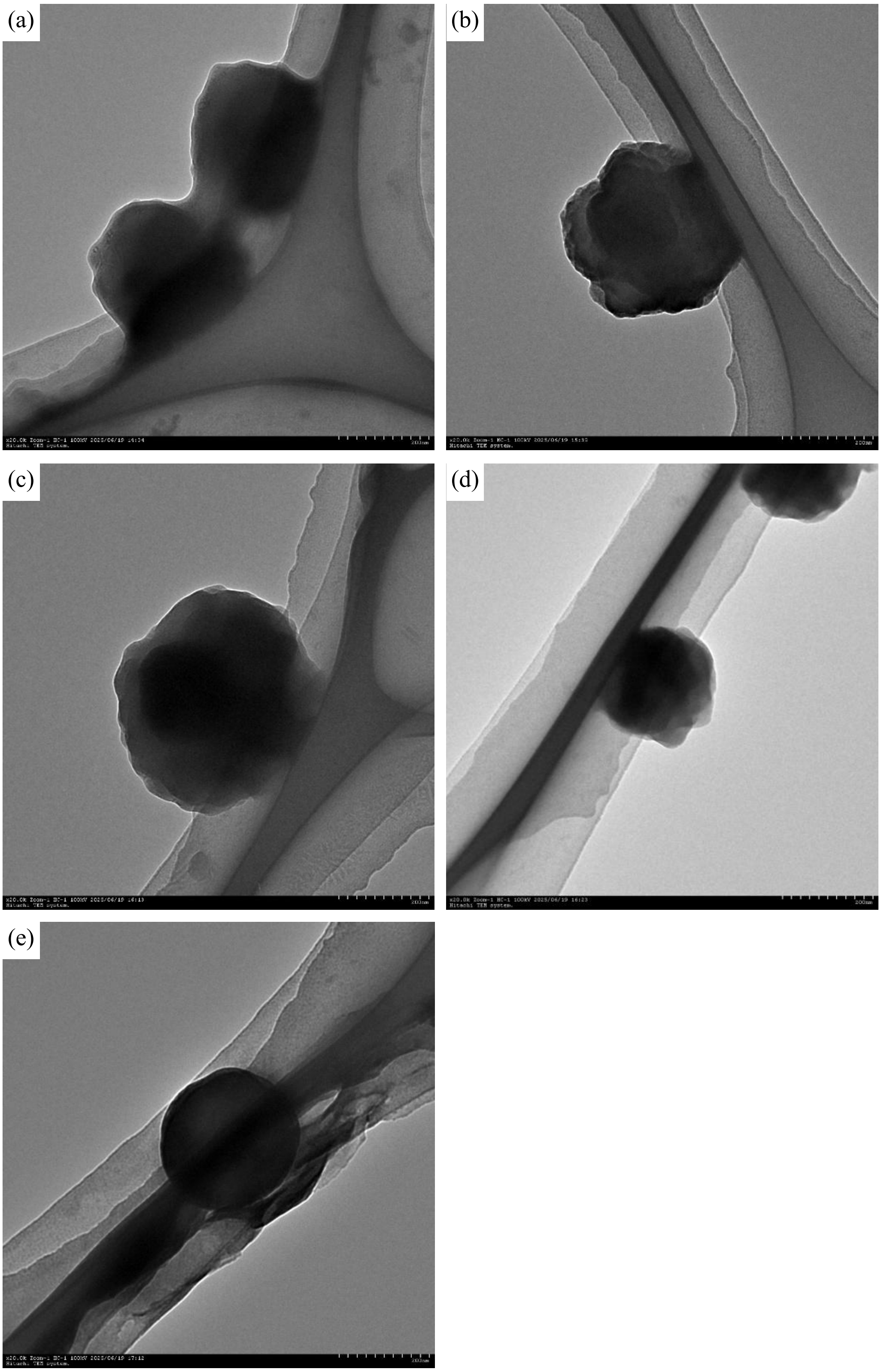

Figure S11 TEM images of dried polymer nanoparticles for (a) entry 1: 320 nm, (b) entry 2: 270 nm, (c) entry 3: 330 nm, (d) entry 4: 250 nm, (e) entry 5: 240 nm. Scale bar: 200 nm.

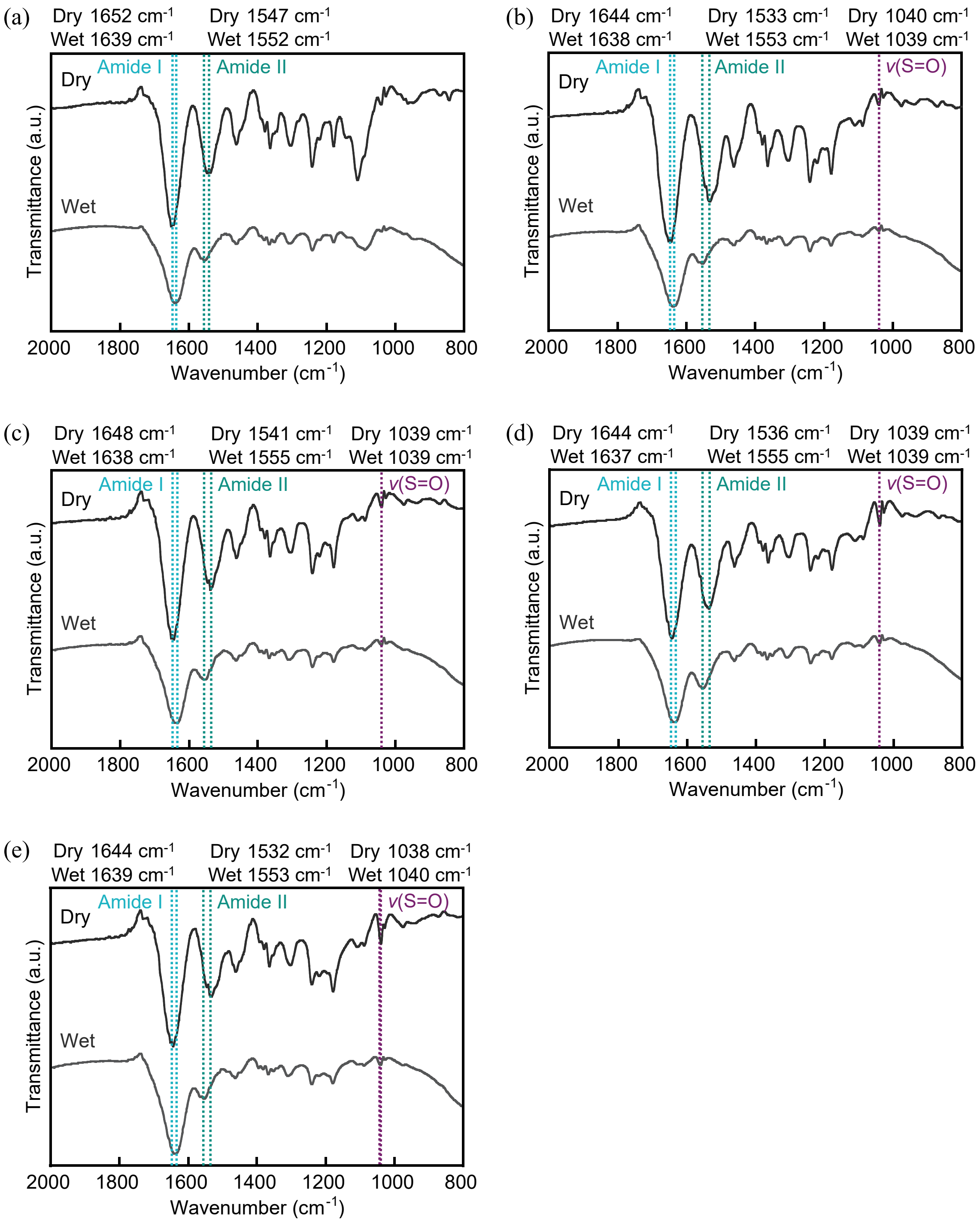

Figure S12 ATR-FTIR spectra of polymer nanoparticles (entry 1 ~ 5). Upper spectra correspond to dried samples, and lower spectra to water-containing samples. Water content (wt%) in the moist samples was as follows: (a) entry 1: 55 wt%, (b) entry 2: 43 wt%, (c) entry 3: 39 wt%, (d) entry 4: 38 wt%, (e) entry 5: 46 wt%. Water content was estimated by TG-DTA.

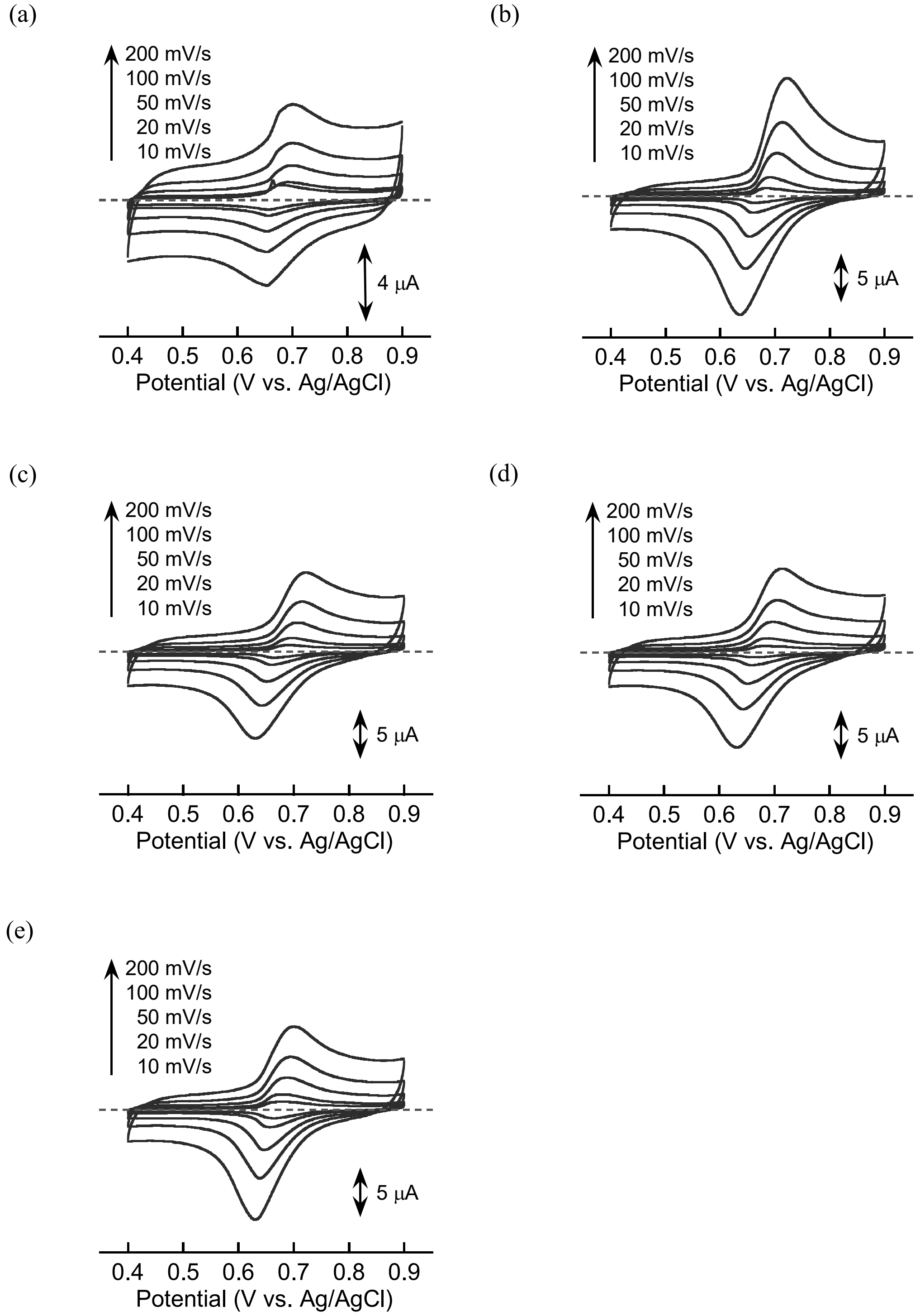

Figure S13 Cyclic voltammogram of 5 mM each nanoparticle dispersion, scanned at 10, 20, 50, 100 and 200 mV/s (a) Entry 1, $y = 0.00$ (b) Entry 2, $y = 0.01$ (c) Entry 3, $y = 0.03$ (d) Entry 4, $y = 0.05$ and (e) Entry 5, $y = 0.10$.

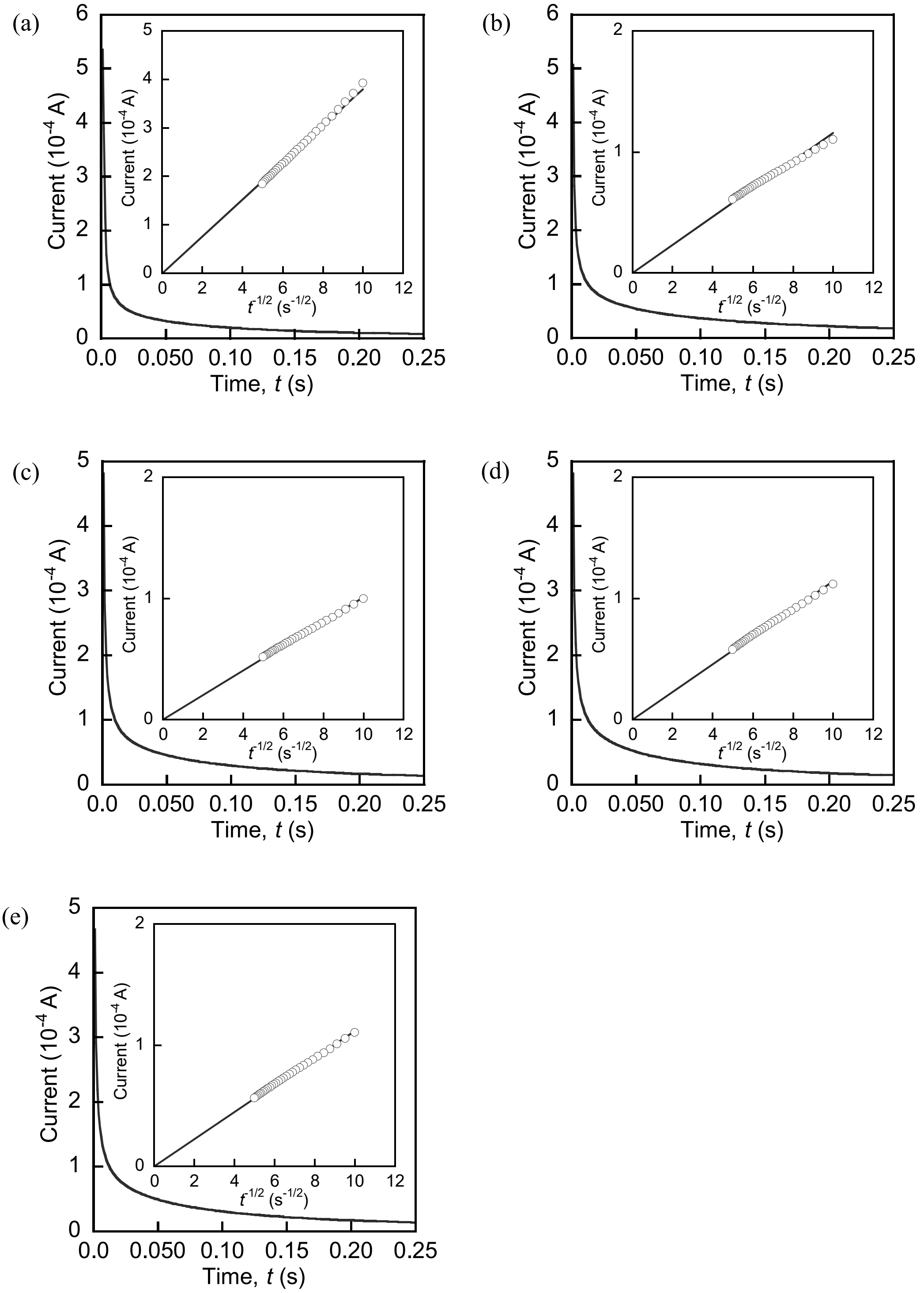

Figure S14 Chronoamperogram of 1 mM each nanoparticle dispersion, potential set to 0.95 V vs. Ag/AgCl, inset: Cottrell plot (a) Entry 1, $y = 0.00$ (b) Entry 2, $y = 0.01$ (c) Entry 3, $y = 0.03$ (d) Entry 4, $y = 0.05$ and (e) Entry 5, $y = 0.10$.

Table S1 Summery of electrochemical parameters for insoluble polymer active materials

Active Materials	Redox group	$E_{1/2}$ V vs. Ag/AgCl	log D_{app} (-)	log k_0 (-)	Reference
Entry 1	TEMPO	0.68	−5.8	−2.3	This work
Entry 2	TEMPO	0.68	−6.0	−2.5	This work
Entry 3	TEMPO	0.68	−6.0	−2.6	This work
Entry 4	TEMPO	0.68	−5.8	−2.3	This work
Entry 5	TEMPO	0.67	−5.9	−2.6	This work
P(VCO−Th)	Thianthrene	1.28 [a]	−7.6	−5.0	[1]
PHQ	Hydroquinone	0.89 [b]	−6.0	−3.2	[2]
PTAm	TEMPO	0.6 [c]	−6.3	N/A	[3]
PTAm/C	TEMPO	0.6 [c]	−5.5	N/A	[3]
PI1	NDI	0.26, 0.095, 0.025, −0.09 [b]	−7.2	−2.6	[2]
PI2	NDI	0.08 [b]	−6.9	−4.0	[2]
PTPM	Viologen	−0.4 [c]	−5.5	N/A	[3]
PTPM/C	Viologen	−0.4 [c]	−5.9	N/A	[3]

[a] Measured in 1 M LiTFSI EC/DEC = 3/7 (v/v) solution, 2 mM active material dispersion. Converted from a standard hydrogen electrode (SHE) to an Ag/AgCl electrode in 3 M NaCl aqueous solution internal standard. Thianthrene is redox-inactive in aqueous electrolyte.

[b] Measured in 2 M H_2SO_4 aqueous solution, 5 mM active material dispersion. Converted from a standard hydrogen electrode (SHE) to an Ag/AgCl electrode in 3 M NaCl solution internal standard.

[c] Measured in 3 M NaCl aqueous solution, 1 mM active material dispersion.

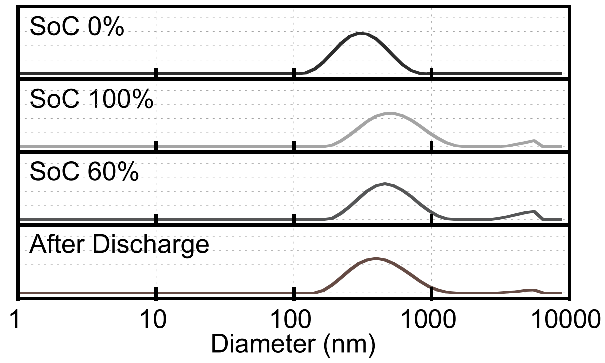

Figure S15 DLS distribution of entry 1 nanoparticles dispersion at each state of charge in 0.1 M NaCl aqueous solution.

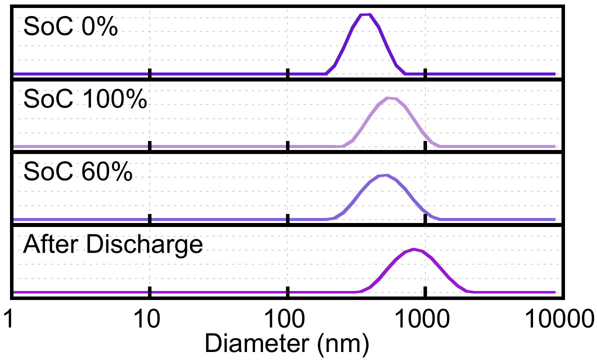

Figure S16 DLS distribution of entry 2 nanoparticles dispersion at each state of charge in 0.1 M NaCl aqueous solution.

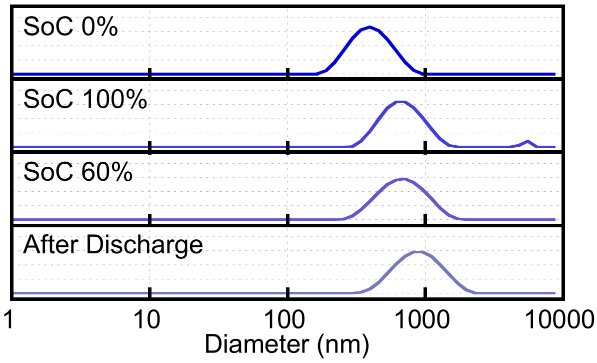

Figure S17 DLS distribution of entry 3 nanoparticles dispersion at each state of charge in 0.1 M NaCl aqueous solution.

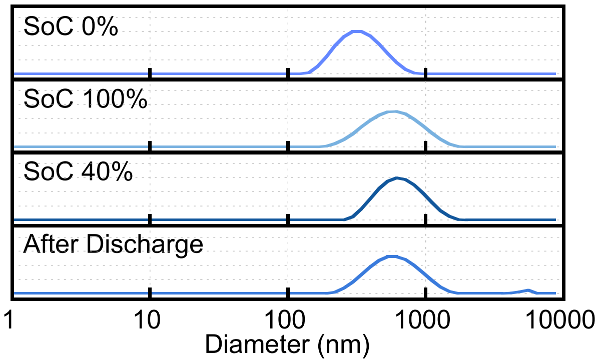

Figure S18 DLS distribution of entry 4 nanoparticles dispersion at each state of charge in 0.1 M NaCl aqueous solution.

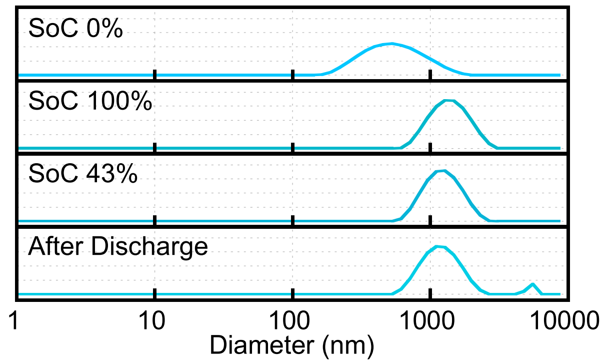

Figure S19 DLS distribution of entry 5 nanoparticles dispersion at each state of charge in 0.1 M NaCl aqueous solution.

Table S2 Particle size and physical diffusion coefficient at each state of charge (SoC)

Entry	Full charged		Intermediate		Full discharged	
	d (10^2 nm)	log D_{app} (-)	d (10^2 nm)	log D_{app} (-)	d (10^2 nm)	log D_{app} (-)
1	5.3	−8.0	4.6 a)	−8.0 a)	4.4	−7.9
2	5.9	−8.0	5.3 a)	−8.0 a)	8.9	−8.2
3	6.9	−8.1	7.2 a)	−8.1 a)	9.4	−8.2
4	6.3	−8.0	7.0 b)	−8.1 b)	6.2	−8.0
5	14	−8.4	13 c)	−8.4 c)	12	−8.4

Intermediate: a) SoC 60 %, b) SoC 40 %, and c) SoC 43 %.

Reference

[1] K. Hatakeyama-Sato, K. Sadakuni, K. Kitagawa, K. Oyaizu, *Sci. Rep.* **2023**, *13*, 5711.

[2] W. Yan, C. Wang, J. Tian, G. Zhu, L. Ma, Y. Wang, R. Chen, Y. Hu, L. Wang, T. Chen, J. Ma, Z. Jin, *Nat. Commun.* **2019**, *10*, 2513.

[3] K. Hatakeyama-Sato, T. Nagano, S. Noguchi, Y. Sugai, J. Du, H. Nishide, K. Oyaizu, *ACS Appl. Polym. Mater.* **2019**, *1*, 188–196.
